# Potential of Aluminum Drug Packages with Press-Through Packaging Considering Usability for a Wide Range of Users

**DOI:** 10.2147/MDER.S482277

**Published:** 2024-11-09

**Authors:** Kiyoshi Kubota, Morio Shimada, Hiroyuki Ura, Kiyomi Sadamoto

**Affiliations:** 1 Department of Clinical Pharmacy, Shonan University of Medical Science, Yokohama, Japan; 2 MECSION, General Incorporated Association, Hiratsuka, Japan; 3 Department of Radiological Sciences, Faculty of Health Sciences, Komazawa University, Tokyo, Japan; 4 Sadamoto Clinic, Kanagawa, Japan

**Keywords:** pharmacists, patients, improved openability, sarcopenia, rheumatoid arthritis

## Abstract

**Purpose:**

Press-through packages (PTPs) are frequently used for the packaging of prescription drugs in Japan. However, tablets or capsules packaged in PTPs may become unstable and difficult to store. Therefore, aluminum pouches (pillow packages) are applied as an additional packaging option. Especially for 10-sheet tablet (capsule) PTPs, there are many opportunities for health-care professionals (mainly pharmacists) to open pillow packages during the dispensing process. However, aluminum pouches (pillow packages) that are easy to open and store appropriately by not only pharmacists, but also patients, are needed.

**Methods:**

A 100-unit PTP (pillow package) with conventional specifications **①** and two other products (**②**: open vertically by a wavy processed half-cut and **③**: pinch the backlining open) were developed with the aim of improving the ease of opening. The study participants, consisting of pharmacists and patients, performed tasks such as opening and taking drugs out of the PTP for each sample, and evaluated the differences in usability.

**Results:**

The results of a sensory test revealed that pharmacists rated products **②** and **③** higher than product **①**. On the other hand, patients, including those with weak grip and pinching strengths, rated **③** highly, confirming the superiority of usability in the order of **③, ②**, and **①**. In addition, item **③** was successfully opened by all patients.

**Conclusion:**

The present results indicated the superiority of the developed pillow packaging, which enables pharmacists to save time in the dispensing process. In addition, product ③ was evaluated highly by patients, especially those with disabilities, for its ease of use not only in terms of opening, but also storage. Sensory testing by actual users applying ergonomic methods enabled a multifaceted evaluation of the products and provided insights into the actual status of pharmacists’ dispensing work (product issues) and patients’ daily medication use.

## Introduction

**Figure 1:**
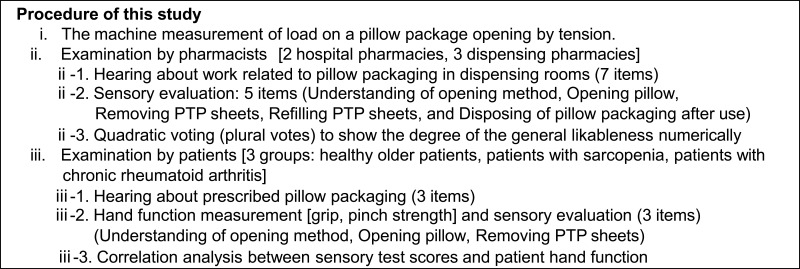
Summary of the procedures of this study.

As a wide variety of people handle pharmaceuticals in their daily lives, ensuring the ease of use and safety of pharmaceuticals is essential in any setting. For example, pharmacists want to provide medicines to patients as quickly as possible, so ease of dispensing is important. In addition, there are various forms of drug delivery in the community, and patients are increasingly managing their own medications over the long term. Therefore, it is essential that medicines be easier and safer to handle for all users, including older adults and patients with hand disabilities.^1^ If pharmaceutical packaging is difficult to handle, it takes time for patients to prepare to take prescription medications.^2^,^3^ Therefore, packaging that is easy to use for both pharmacists and patients is needed.^4^ This study aimed to compare the usability of three different sample designs using pillow packaging, which is commonly employed to ensure the stability of press-through package (PTP) products.^5^ The three types compared are a conventional specification and two trial products developed to improve the ease of opening. Figure 1 provides a summary of the procedures of this study.

## Materials and Methods

### Evaluation Samples

**Figure 2:**
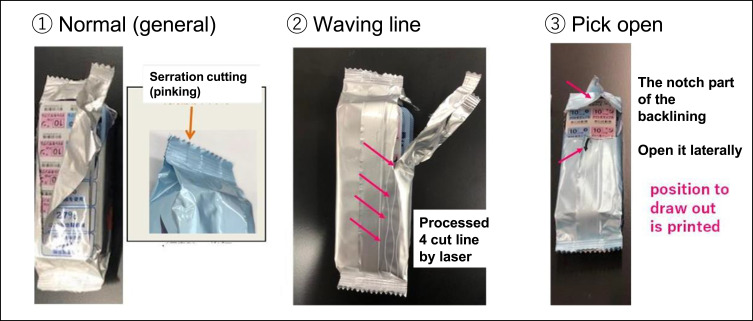
The three types of pillows and appearances after opening.

In this study, 100-unit PTP sheets (size: 95×45 × 20 mm) and aluminum pillow packages (size: 125×49 × 25 mm; with a three-way seal characterized by low porosity and compactness) were used. The film material was composed of PET 16/dry laminate/AL 20/PE 30. The appearance of the three samples, described as follows, is shown in Figure 2: **①** is general pack: starting from the serration, we cut the pillow package lengthwise; **②** is referred to as a wavy line pack: we cut the pillow package along a line processed by a laser lengthwise;^6^ and **③** is referred to as a pick open pack: we opened the package using a notch on the backlining and opened it laterally.

### Study Methods

We conducted a comparative evaluation of the three types of aluminum pouches (pillow packages) in terms of suitability of use, including the suitability for dispensing work. The three samples were **①** a general packaging type currently in use, and two newly developed packages designed for improved opening and storage (sample **②** was packaging with vertical stripes for improved opening), and sample **③** was packaging that can be opened using a pull-up method).^7^ Pharmacists and patients who actually handle these products were recruited to evaluate these samples. First, the amount of clamp movement and the force applied to the clamp (force required to tear) were measured in order to compare the characteristics related to the openability of the three sample types.^8^

Pharmacists were asked seven questions about pillow packaging and dispensing operations, followed by a five-item sensory evaluation test, including opening. In addition, pharmacists were asked to vote on their overall preferred sample as a packaging form to be handled by pharmacists. This voting is called multiple voting (quadratic voting [QV]), which allows multiple votes for multiple candidates.^9^

Next, three groups were selected for evaluation: a group of healthy older adult subjects without hand disorders, a group of older adult subjects with sarcopenia (weak hand strength), and a group of patients with rheumatoid arthritis (RA). Prior to sensory testing, three questions about the pillow packaging were asked, and the subjects’ grip and pinch strength were also measured.^10–12^

The human trials in this study are clinical trials. Therefore, the REC and IRB are being conducted at Sadamoto Clinic by the following Ethics Committee Members (Proposal, Consent Form, Approval Form No. YM20210305). In addition, although there is no risk of health damage or stress during the trial, informed consent is provided to the pharmacists and patients who are the subjects of the trial. These clinical trials were conducted by the author, Kiyomi Sadamoto (a specialist in rheumatology) from May to December 2021.

Ethics Committee Member: Takaharu Sadamoto[Chairman; Clinic Director], Hiromi Matayoshi [head nurse], Masaho Hayashi[Pharmaceutical Packaging Specialist)], Yuji Morio[MD], Shinya Aoki[Caregiver], Kazumi Shimohira[Caregiver], These committee members have agreed to have their names and occupations published in this paper.

## Instruments

### Pulling Strength Measurement

**Figure 3:**
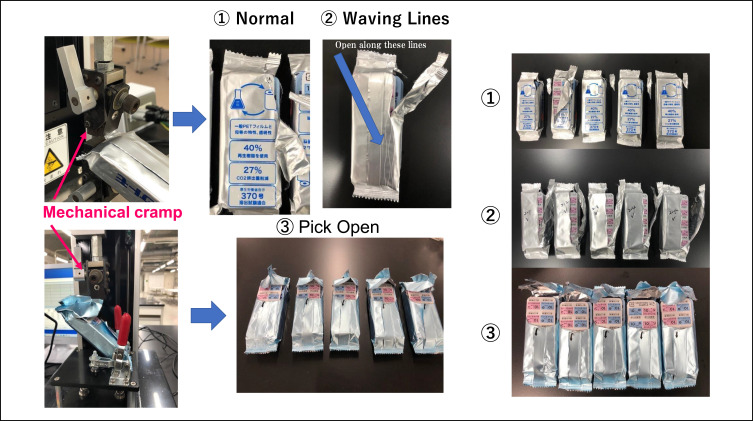
Appearance of tensile tester and the three types of pillows after testing.

The Force Tester MCT-1150/2150 (A&D Company, Tokyo, Japan) was used to measure pulling strength. Draw the travel distance of the Force Tester’s crosshead (clamp) connected to the load cell and the force to tear the pillow package.^13^ The mechanical clamp and the appearance of the three pillow types after the tensile tear test are shown in Figure 3. The measurement conditions and name of the software used in the data analysis were as follows:

Load cell: 500 N

Test force indication separation capacity: 0.1 N

Crosshead *mode*: tension, *stroke*: 150 mm, *speed*: 50 mm/min

Data processing software: MSAT-Light (A&D Company)

### Hand Function Measurement

Grip and pinch strength were measured using the following instruments to assess patient hand function:

Grip strength: Smedley-type Hand Dynamometer TKK5401 [100 kg] (Takeikiki Kogyo, Niigata, Japan)

Pinch strength: Isoforce GT-300 [100 N] (OG Giken, Okayama, Japan)

### Commentary Board for NRS Sensory grading^14^

Subjects perform and score sensory tests with reference to the following board. This board contains a guide to the items on the test and the corresponding grades.

Evaluated items ※1, and score (NRS)

NRS evaluation / 10-point grading system using five terms^15^

**Table:** 

**NRS**: Five terms and their corresponding scores Extremely poor ⇔ Somewhat poor ⇔ Neither good nor poor ⇔ Somewhat good ⇔Extremely good 1,2 3,4 5,6 7,8 9,10

※1 The evaluation criteria were as follows. Pharmacists: understanding of the opening method, ease of opening the pillow package, ease of removing the PTP from the pillow package, ease of refilling the PTP into the pillow package, and ease of disposal of the used pillow package. Patients: understanding of the opening method, ease of opening the pillow package, ease of removing the PTP from the pillow package.

### Statistical Analysis

Excel Ver. 2312 [build 17126, 20132] in Microsoft 365

(Microsoft Corporation, Redmond, Washington, USA)

Statistical summary values: mean, standard deviation

Bar graph display of the distribution of survey responses

Test of the difference of means (between four patient groups and three pillow types)

Correlation coefficient (NRS vs patient status: grip strength, pinch power)

## Results

### Instrumental Measurements

The opening characteristic test was repeated five times using a tensile tester (force tester) and the characteristics of each sample were measured. The following three parameters were used as characteristic values (Table 1): maximum tensile force, distance to the maximum tensile force point, and weight change of the detached package pieces. Table 1 shows that there was little difference in the maximum load among the three samples. In addition, sample **③** required slightly more force to start cutting the pillow open with light force. In addition, **③** was cut apart in a uniform manner with little unevenness in the weight of the cut film. On the other hand, in **①**, the cutting load gradually increased throughout the opening stroke. For **②**, the opening process was basically the same as that for **①**, but because it was cut open along the half-cut line, there was no variation as in **①**.

**Table 1:** Opening Characteristics of the Pillows Measured by Force Tester

Measurement Item	① Normal	② Wavy line	③ Pick open
**Maximum load** [n = 5 Mean/SD]	9.2 / 1.0 N	11.5 /**2.6**	9.0 /1.2
**Moving quantity to maximum load point** [n= 5 Mean]	29.2 mm	35.0	**9.0**
**Weight of the cutting film** ※ [n = 5 Mean / SD]	130 / 140 mg	430 / 50	350 / **10**

**Notes**: ※ Each sample that has not been detached is cut and weighed at the end point of the tear. The values in bold and underlined indicate values that differ from other samples.

### Examination by Pharmacists

The examination by pharmacists involved 20 participants (male: female ratio = 7:13). The experiment consisted of seven hospital pharmacists from two facilities and 13 pharmacy pharmacists from three facilities. The pharmacists’ mean experience was 15.6 ± 13.6 years (range, 1–43 years).

#### Knowledge About Work Related to Pillow Packages

**Figure 4:**
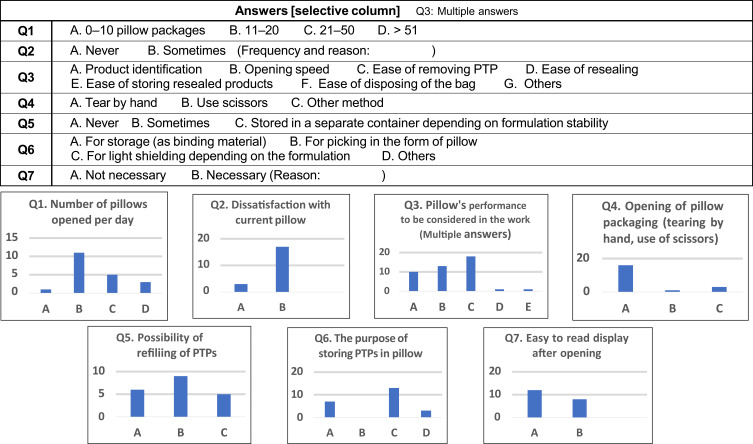
Items heard about by pharmacists regarding pillow packaging.

Prior to the sensory test, questions were asked regarding the amount of pillow packages handled in pharmacy operations, dissatisfaction with and requests for current specifications, storage methods after opening, and pillow package labeling (Table 2). The results are shown in Figure 4. The pharmacists requested that the pillow be opened quickly and that the PTP be removed quickly. The purposes for refilling to the pillow were binding (60%) and shading (40%). In addition, 80% of the subjects opened 11–50 pillows per day, 15% were dissatisfied with current pillow packaging, and only 5% opened pillows with scissors (most used their fingers). Moreover, 30% of the subjects never refilled PTPs after opening, 45% refilled PTPs in the pillow, 25% stored them in a separate container for stability, and 35% required an easy-to-read pillow display after opening.

**Table 2:** Items Heard About by Pharmacists Regarding Pillow Packaging

Question Items	
**Q1**	Number of pillows opened per day in daily operations: Number of pillows to be opened
**Q2**	Have you ever been dissatisfied with the pillow packaging?: Dissatisfaction with current pillow packaging
**Q3**	In terms of ease of handling, the factors you are looking for are [multiple answers allowed]: Ease of use factors
**Q4**	How to open the package: Opening method
**Q5**	Do you store PTPs in the pillow after opening?: Possibility of refilling of PTPs
**Q6**	What is the purpose of storing PTPs in the pillow after opening?
**Q7**	Is it necessary to have an easy-to-read display on the pillow after opening?

#### Sensory Test for Pharmacists

**Figure 5:**
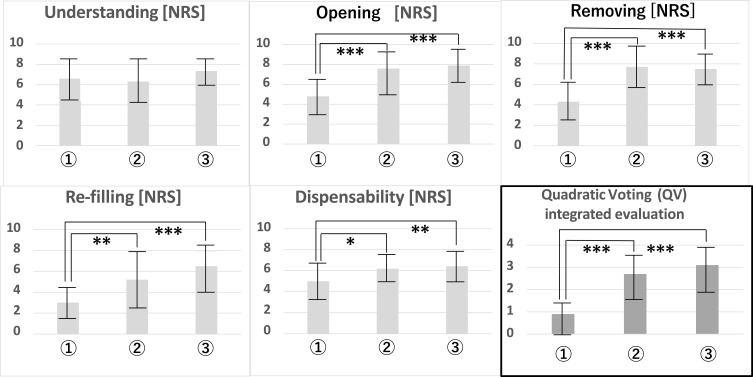
Results of Student’s *t*-test for sensory test and QV voting by pharmacists. Significance level: *5%, **1%, ***0.1%.

The pharmacists opened the samples, removed the PTP, and refilled the PTP in the pillow packages. They then scored the following five items as a sensory evaluation: understanding of the opening method, ease of opening the pillow, ease of removing the PTP from the pillow, ease of refilling the PTP into the pillow, and ease of disposal of the used pillow package. Finally, a vote (QV※2) was conducted on the sample that was considered to be the most desirable overall. Table 3 summarizes the results of the sensory evaluation scores (NRS) and QV for each pillow sample. The results of a Student’s *t*-test between pillow samples are summarized in Figure 5. No differences in understanding how to open the pillow were found among samples. Very clear differences in openability were found, with **②** and **③** being easier to open. The results regarding ease of opening and PTP removal from the pillow were similar. Refill ability was also clearly better in **②** and **③** than in **①**. Disposal of used pillows was also better in **②** and **③** than in **①**. The overall preference for samples was in the order of **③, ②, ①**. The number of votes for **①** was quite low, whereas 14 of 20 panel members preferred **③** the most.

※2 This decision-making policy (QV) expedites efficiency as the number of voters increases. The simplified formula on how QV functions is as follows:

**Table 3:** Sensory Test Results and QV Voting of Pharmacists [Shading and Bold: Most Votes by QV]

Years of Experience		Sex	Understanding			Opening			Removing PTP			Refilling PTP			Disposability			QV: Overall Desirability		
			①	②	③	①	②	③	①	②	③	①	②	③	①	②	③	①	②	③
**1**	30	FM	4	4	7	4	4	7	2	2	7	2	2	3	5	5	5	0	3	**4**
**2**	8	FM	5	7	7	5	6	8	5	8	5	5	4	4	5	5	5	1	2	3
**3**	23	FM	5	3	9	5	9	9	3	10	7	1	1	7	5	5	5	0	4	3
**4**	37	FM	5	5	6	1	7	6	1	7	6	1	7	6	1	6	6	0	4	3
**5**	8	M	9	5	8	4	7	9	2	5	10	3	7	9	4	8	8	0	2	4
**6**	43	M	7	8	7	7	8	6	6	7	8	4	6	4	9	9	9	3	2	3
**7**	41	FM	7	6	5	6	6	7	5	7	8	2	6	7	8	8	8	2	3	3
**8**	1	FM	4	5	9	3	9	9	3	6	8	5	5	5	5	5	5	0	3	4
**9**	1	FM	8	8	7	7	9	9	7	10	10	4	8	9	4	7	8	0	3	4
**10**	2	FM	5	7	9	4	9	8	7	10	8	4	8	9	6	6	6	0	3	4
**11**	18	M	6	8	7	3	9	10	3	8	7	5	3	7	4	8	8	0	2	3
**12**	8	FM	6	8	8	5	9	9	4	9	9	3	9	9	5	5	5	0	3	4
**13**	2	FM	5	7	7	4	9	9	3	9	7	2	8	7	5	6	7	0	3	4
**14**	10	FM	6	5	4	4	7	9	4	6	5	2	2	7	6	5	5	2	0	4
**15**	6	FM	5	3	8	7	3	7	6	6	7	2	3	4	7	7	8	3	0	3
**16**	6	FM	6	3	7	5	6	6	5	9	6	5	2	2	4	6	6	3	3	0
**17**	16	M	10	10	6	8	9	10	8	8	10	4	4	10	5	5	5	1	2	4
**18**	7	M	4	8	8	2	8	8	2	9	9	1	9	4	5	5	5	1	4	2
**19**	20	M	10	9	8	5	8	3	5	8	6	3	7	6	4	5	5	1	3	1
**20**	25	M	10	7	7	6	9	8	5	9	7	2	3	7	2	8	8	0	4	2
**Mean**			6.4	6.3	7.2	4.8	7.6	7.9	4.3	7.7	7.5	3.0	5.2	6.3	5.0	6.2	6.4	0.9	2.7	3.1

Cost to the voter = (Number of votes) ^ 2 = credits to be given to the panel [eg, 25]

### Examination by Patients

Regarding the examination by patients, their characteristics were as follows:

Participants: 26 (male: female ratio = 5:21; mean age: 68.0 ± 11.1 years)

Healthy older adults: 10 (male: female ratio = 4:6; mean age: 65.5 ± 12.5 years)

Patients with sarcopenia ※3: 8 (male: female ratio = 1:7; mean age: 67.5 ± 11.0 years)

Patients with RA ※4: 8 (male: female ratio = 0:8; mean age: 71.8 ± 9.8 years)

※3 Grip strength: F < 18 kg, M < 26 kg (defined by Asian consensus)^16^

※4 Chronic RA with deformity, stiffness, and pain in the fingers

#### Knowledge About Pillow Packages

Prior to the sensory test, three questions were asked about the pillow packages. The participants were divided into four groups to compile responses. As shown in Table 4, we found that three out of four patients had experience with prescriptions in pillow packages (about 90% of patients with chronic RA), about 40% of the panel used scissors to open the pillow packages (about 30% of healthy seniors and patients with RA), and about 50% of panels stored PTPs back in the pillow package.

**Table 4:** Patients’ Hearing Results (by Group)

Group	Members of the Panel	Q1. Experienced in Prescribing Pillows	Q2. How to Open the Pillows		Q3. PTP Storage Method After Opening Pillows		
			By Hands	By Scissors	Open	Into Pillow	Another Container
**W**	**All patients**	73%	63%	37%	32%	52%	16%
**A**	**Healthy seniors**	60	67	33	17	50	33
**B**	**Patients with sarcopenia**	75	50	50	33	67	0
**C**	**Patients with RA**	88	71	29	43	43	14

#### Sensory Test by Patients

Prior to the sensory test, grip strength and pinch power were measured in the patients. Sensory tests were used to evaluate the characteristics of each sample, including understanding how to open, ease of opening, and ease of removing the PTP from the pillow package (Table 5).

**Table 5:** Sensory Test Results of Patients [Shading: Sample with the Highest Grade]

	Subject Group	Patient Status			Understanding			Opening Pillow			Removing PTP		
		Sex	Grip Strength	Pinch Strength	①	②	③	①	②	③	①	②	③
**1**	**A**	FM	24.3 kg	55 N	4	7	9	3	7	8	–	–	–
**2**	**Healthy seniors**	M	32.5	94	6	6	10	8	8	8	–	–	–
**3**		FM	26.6	44	3	7	8	4	6	8	–	–	–
**4**		FM	23.7	61	5	10	9	0	10	9	–	–	–
**5**		M	35.8	48	5	6	8	4	6	8	–	–	–
**6**		FM	20.7	41	4	5	8	5	6	8	3	5	8
**7**		M	39.6	100	5	5	9	5	5	9	5	3	9
**8**		FM	20.7	70	4	7	8	4	8	9	3	7	8
**9**		FM	20.1	47	7	8	10	7	8	10	4	8	10
**10**		M	46.2	84	4	8	8	3	8	8	3	8	9
**Mean**			**29.0**	**64.4**	**4.7**	**6.9**	**8.7**	**4.3**	**7.2**	**8.5**	**3.6**	**6.2**	**8.8**
**11**	**B**	FM	**14.2**	41	8	6	6	8	5	6	–	–	–
**12**	**Patients with sarcopenia**	FM	**16.2**	17	5	5	7	5	5	8	–	–	–
**13**		M	**24.8**	72	3	7	9	7	7	9	–	–	–
**14**		FM	**17.3**	28	4	4	8	6	8	8	6	6	8
**15**		FM	**17.1**	45	8	6	10	3	8	10	6	8	10
**16**		FM	**15.4**	40	5	1	4	5	0	4	6	0	4
**17**		FM	**17.2**	51	5	6	8	7	7	8	7	7	9
**18**		FM	**14.6**	35	6	7	9	6	7	8	6	7	9
**Mean**			**17.1**	**41.1**	**5.5**	**5.3**	**7.6**	**5.9**	**5.9**	**7.6**	**6.2**	**5.6**	**8.0**
**19**	**C**	FM	12.9	38	4	5	6	4	4	5	–	–	–
**20**	**Patients with rheumatoid arthritis**	FM	0.0	0	4	5	8	2	2	3	–	–	–
**21**		FM	9.5	41	4	5	9	7	7	7	–	–	–
**22**		FM	10.5	49	7	7	9	7	7	9	8	8	10
**23**		FM	10.5	30	7	8	9	7	8	9	7	8	9
**24**		FM	0.0	25	2	1	6	2	0	6	2	0	6
**25**		FM	14.5	49	7	7	8	7	7	8	7	7	8
**26**		FM	14.6	60	4	8	10	3	8	10	3	7	10
**Mean**			**9.1**	**36.5**	**4.9**	**5.8**	**8.1**	**4.9**	**5.4**	**7.1**	**5.4**	**6.0**	**8.6**
**All patients**		**Mean**	**19.2**	**48.7**	**5.0**	**6.0**	**8.2**	**5.0**	**6.2**	**7.8**	**5.1**	**5.9**	**8.5**

**Figure 6:**
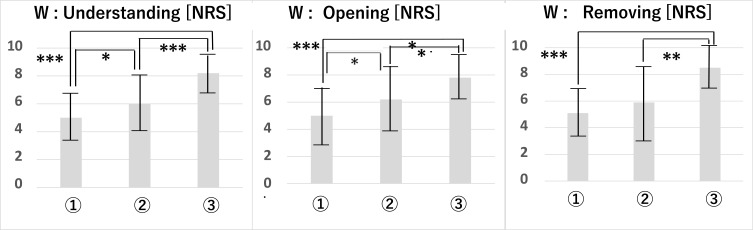
Student’s *t*-test results for the sensory tests by all patients. Significance level: *5%, **1%, ***0.1%.

From these data, Student’s *t*-tests were performed between patient groups (Table 6) and pillow samples (Table 7). The results for all patient data are shown in Figure 5. From Tables 6 and 7 and Figure 6, we found that the characteristics of each patient group were well represented in regard to grip and pinch strength. Only A (healthy seniors) and B (sarcopenia) in PTP removing test showed significant differences in Student’s *t*-test between each patient group.

**Table 6:** Student’s *t*-Test Results for the Sensory Test Between Patient Groups

Pillow Sample	Subject Group (Patient group)	Student’s *T*-Test of Nrs Scores for the Sensory Evaluation (3 Items) Between Groups								
		Understanding (Opening pillow)			Opening Pillow			Removing PTP from Pillow		
		A	B	C: RA	A	B	C: RA	A	B	C: RA
**①** **Normal** **pillow**	W:: Whole patients	0.54	0.49	0.87	0.43	0.20	0.93	0.52	0.51	0.81
	A:: Healthy seniors		0.29	0.82		**0.096^※^**	0.61		0.001**	0.12
	B:: Sarcopenia			0.51			0.34			0.55
**②** **Wavy** **line pillow**	W:: Whole patients	0.18	0.35	0.76	0.16	0.74	0.48	0.83	0.84	0.97
	A:: Healthy seniors		**0.07^※^**	0.25		0.23	0.15		0.74	0.91
	B:: Sarcopenia			0.65			0.73			0.85
**③** **Pick open** **pillow**	W:: Whole patients	0.20	0.46	0.91	**0.099^※^**	0.81	0.47	0.56	0.70	0.88
	A:: Healthy seniors		0.17	0.34		0.24	0.15		0.50	0.82
	B:: Sarcopenia			0.57			0.64			0.66

**Notes**: The bold values are statistically significant or have a significant trend. Significance level ** 1%, **^※^** Significant difference trend.

**Table 7:** Student’s *t*-Test Result for the Sensory Test Sensory Test Between Pillow Samples

Subject Group (Patient Group)	Pillow Sample	Student’s *t*-Test of Nrs Scores for the Sensory Evaluation (3 Items) Between Groups					
		Understanding		Opening Pillow		Removing PTP	
		②	③: POP	②	③: POP	②	③: POP
**W**: **Whole patients**	**①: Normal**	0.04 *	0.000 ***	0.050 *	0.000 ***	0.33	0.000 ***
	**②: Wavy line**		0.000 ***		0.010 **		0.006 **
**A**: **Healthy seniors**	**①: Normal**	0.002 **	0.000 ***	0.003 **	0.000 ***	0.053**^※^**	0.000 ***
	**②: Wavy line**		0.005 **		0.03 *		0.053**^※^**
**B**: **Sarcopenia**	**①: Normal**	0.79	0.04 *	1.00	0.06**^※^**	0.70	0.16
	**②: Wavy line**		0.03 *		0.15		0.22
**C**: **Rheumatoidarthritis**	**①: Normal**	0.42	0.002 **	0.72	0.08**^※^**	0.77	0.06**^※^**
	**②: Wavy line**		0.03 *		0.22		0.18

**Notes**: Significance level * 5%, ** 1%, *** 0.1%, **^※^** Significant difference trend.

The results of Student’s *t*-test between pillow samples showed different strengths of significant differences for each patient group. Ease of understanding of openability was rated lowest for **①**, medium for **②**, and highest for **③**. This trend was the same across patient groups. No statistically significant differences were found between samples in pillow opening or PTP removing test between the sarcopenia and RA groups. On the other hand, a significant difference was seen in the healthy seniors, with **①** being inferior to **②** and **③** (a comparison between **②** and **③** showed that **③** was significantly higher).

#### Relationship Between Patient Status and Ease of Opening and PTP Removal

From the data in Table 5, we calculated the correlation coefficients for grip and pinch strength for the three patient groups with NRS scores for each sample in regard to the ability to open the pillow and remove the PTP. Table 8 shows the correlation coefficients between patient status and NRS scores.^17^ The results showed high positive correlations between grip and pinch strength and ease of opening and removing PTPs for the patients with RA.

**Table 8:** Correlation Coefficients Between Patient Status and NRS Scores

Patient Group	Evaluation Item×Patient Status		① Normal Pillow	② Wavy Line	③ Pick Open Pillow
**W**: **All** **patients**	**Ease of opening**	**Grip strength**	−0.02	0.40	0.41
		**Pinch strength**	0.11	0.44	0.49
	**Removing PTPs**	**Grip strength**	−0.21	0.18	0.24
		**Pinch strength**	−0.23	0.11	0.29
**A**: **Healthy** **seniors**	**Ease of opening**	**Grip strength**	−0.01	−0.25	−0.36
		**Pinch strength**	0.19	0.06	0.02
	**Removing PTPs**	**Grip strength**	0.24	−0.15	0.20
		**Pinch strength**	0.47	−0.36	0.05
**B**: **Patients with sarcopenia**	**Ease of opening**	**Grip strength**	0.13	0.32	0.47
		**Pinch strength**	0.31	0.16	0.21
	**Removing PTPs**	**Grip strength**	0.40	0.39	0.38
		**Pinch strength**	**0.71**	0.14	0.19
**C**: **Patients with rheumatoid** **arthritis**	**Ease of opening**	**Grip strength**	0.59	**0.84**	**0.67**
		**Pinch strength**	0.46	**0.71**	**0.82**
	**Removing PTPs**	**Grip strength**	0.50	**0.88**	**0.77**
		**Pinch strength**	0.16	0.59	**0.72**

**Notes**: Bold and Shading Value: Correlation coefficient > 0.7.

## Discussion

Some products are packaged in aluminum pillow wrappers, which are secondary packaging, to ensure the stability of PTP pharmaceuticals. The shape of the package, in which the long side of the contents is sealed before the short side, is called pillow packaging because it resembles a pillow. While the contents can be packaged tightly, the film material, combined with its firmness, makes it difficult to open the package. Normal packaging is opened by cutting from the valley of the jagged teeth on the short side, but this requires finger strength, or the contents cannot be removed because they are torn off at an angle. To solve these problems, two types of packaging were developed in the present study: **②**, a type with a corrugated scratch on the film so that it can be cut straight through with little force (wavy line pillow), and **③**, a type with a notch on the long side seal and a scratch on the film around the body (pick open pillow).

First, to obtain the surrogate characteristic of openability, changes in crosshead travel and load were compared by tearing a load cell with a tensile device. Table 1 shows that although there was no difference in the maximum load value among the three samples, there was a large difference in the location of the maximum load and the size of the separated film and its variation. In sample **③**, there were two characteristics: the maximum load was at the initial stage and the area (shape) of the detached film was uniform. In addition, the uniform shape and large opening of the separated pillow packaging made it easy to store the contents again (PTP sheets).

Because it is difficult for laboratory staff with no dispensing experience or knowledge of the actual patient population to evaluate these samples, sensory testing was conducted with pharmacists and older patients to determine the type of product truly desired based on the results of a 3- or 5-item evaluation. Table 2 and Figure 3 show that each pharmacist had different experience levels, working conditions, and opinions about current products. Even among these pharmacists, the developed products **②** and **③** received higher scores than the current pillow **①** in all four sensory evaluations, with the exception of the ease of understanding the opening method.

From the votes obtained by QV, the degree of overall favorability was clear (**②** and **③** were preferred), and the shading in Table 3 shows that there was a difference in the maximum number of votes for **②** and **③**, indicating more support for **③**. As for the reasons for the differences in **②** and **③**, the predominance of the score for ease of extraction was remarkable.

Participants without any literacy (NRS scoreboard comprehension) or cognition problems were recruited for this study. For patients with RA, strict functional assessment (eg, Health Assessment Questionnaire) was omitted, but patients with Larsen Grades 2–4 and Steinbrocker Stage 3 were included, with a rheumatologist serving as coordinator of the sensory study.^18^

From Table 5, it is clear that the patient panel gave higher grades in the order of **③** and **②**. In addition, many of the post-test free comments indicated that **③** was superior in terms of storability, which is well understood as the opinion of patients who need to store and manage their drugs for a long period of time.

The results of Student’s *t*-test between samples for each patient group were omitted because they were almost identical to those for all patients. Table 6 shows that there was a strong correlation between the hand function of patients with RA and the sensory evaluation scores for **②** and **③** in terms of the ability to open the pillow and take out the PTP, which indicates that sensory scores are not merely subjective, but rather, are related to the physical functioning of the panelist. In this sensory test, the patients with RA were highly detectable.

While existing studies using pharmacist subjects have asked questions such as the time it takes to open a pillow and whether the pharmacist prefers the current or developed product, we recommend that a more promising method is to set up detailed sensory evaluation items and conduct numerical analysis using an NRS or other methods. In addition, because the background characteristics of patient panels are diverse, grouping patients together may yield more interesting results (eg, the correlation between pinching force and NRS scores). Therefore, ergonomic studies may be the best way to focus on and understand the reality of the users.

## Conclusion

This study analyzed the usability of aluminum pillow packages, commonly used by pharmacists and patients. Three types of pillow packages were used: one conventionally used and two newly invented. These three types were evaluated in human ergonomic studies by pharmacists and patients, including those with disabilities in their hands. The results showed that both of the new types of pillow packages received significantly better scores compared with the conventional type. In addition, particularly the pull-up type, which is characterized by not only easier opening, but also for stored PTPs, was highly evaluated by patients. These findings indicate that ergonomic and patient preference studies can provide reliable evaluations of usability and contribute to daily adherence among patients.

## Data Availability

All data presented in this paper were obtained from experiments conducted by the authors; no other sources of data are available.
